# Supplementary material for: A 3D Surface Plot for the Effective Visualization of Specific Serum Antibody Binding Properties
Source: Antibodies (Basel). 2025 Aug 13;14(3):68. doi: 10.3390/antib14030068 (PMC12372043; doi:10.3390/antib14030068)
Supplement: Supplementary file 1 [file antibodies-14-00068-s001.zip › persp_3D_play_fin_v1.nb.html]

Antibody binding 3 concentration display


Code 

- Show All Code
- Hide All Code
- Download Rmd

# Antibody binding 3 concentration display

# Aims

- Visualization of antibody binding from serum

# Analyis

Function for visualization:


```
draw_persp_3d <- function (logC, nu, logxi, lower_z, upper_z){
     
 ## Draw surface plot
     # x-axis: antibody concentrations (log[Ab])
     # y-axis: titrating binding sites immobilized on the surface (log[Ag])
     # z-axis: concentrations of bound antibodies (log[Ab]b)
     #par(cex = 0.8)
     par(cex.axis = 0.7,  # smaller tick labels
               cex.lab  = 0.9) 
     x <- seq(-1, 0, length.out = 20)
     y <- seq(-6, -4, length.out = 20)
     z_surface <- outer(x, y, 
                        function(a, b) logC + nu * log((1 / nu) / (1 + exp(-(a)))) / (1 - nu) + log((1 + nu) / (1 + nu * exp(-(b - logxi)))) / nu)
     z_surface[z_surface < lower_z | z_surface > upper_z] <- NA 
     pmat <- persp(x, y, z_surface,
                   col = "grey99", theta = 45, phi = 40, ticktype = "detailed",
                   zlim = c(lower_z, upper_z),
                   xlab = "log[Ab]", ylab = "log[Ag]", zlab = "log[Ab]b",
                   border = "grey70")
     
 ## Red line indicates the relative concentration (log([Ab]f/KD))
     x_highlight <- -log(1 / nu - 1, base = 10)
     z_highlight <- outer(rep(x_highlight, length(y)), y,
                          function(a, b) logC + nu * log((1 / nu) / (1 + exp(-(a)))) / (1 - nu) + log((1 + nu) / (1 + nu * exp(-(b - logxi)))) / nu)[1, ]
     z_highlight[z_highlight < lower_z | z_surface > upper_z] <- NA
     projected_line <- trans3d(x_highlight, y, z_highlight, pmat = pmat) 
     lines(projected_line$x, projected_line$y, col = "red", lwd = 3)
     
 ## Purple line indicates epitope density (log[Ab]b)
     x_line <- rep(min(x), length(y))
     z_line <- rep(logC, length(y))
     projected_line_edge <- trans3d(x_line, y, z_line, pmat = pmat)
     lines(projected_line_edge$x, projected_line_edge$y, col = "purple", lwd = 3)
     
 ## Blue line indicates the affinity (log(KD))
     z_highlight_y <- outer(x, rep(logxi, length(x)),
                            function(a, b) logC + nu * log((1 / nu) / (1 + exp(-(a)))) / (1 - nu) + log((1 + nu) / (1 + nu * exp(-(b - logxi)))) / nu)[, ]
     projected_line_y <- trans3d(x, rep(logxi, length(x)), z_highlight_y[, 1], pmat = pmat)
     lines(projected_line_y$x, projected_line_y$y, col = "blue", lwd = 3)
}
```


Surface display for the characterization of antibody binding to SARS-CoV-2 spike protein in serum


```
draw_persp_3d(logC = -8,
              nu = 0.3,
              logxi = -5,
              lower_z = -11,
              upper_z = -5)
```

LS0tDQp0aXRsZTogIkFudGlib2R5IGJpbmRpbmcgMyBjb25jZW50cmF0aW9uIGRpc3BsYXkiDQpvdXRwdXQ6IA0KICBodG1sX25vdGVib29rOiANCiAgICBjb2xsYXBzZWQ6IHllcw0KICAgIGZpZ19jYXB0aW9uOiB5ZXMNCiAgICBmaWdfaGVpZ2h0OiA0DQogICAgZmlnX3dpZHRoOiA1DQogICAgdGhlbWU6IHNhbmRzdG9uZQ0KICAgIHRvYzogRkFMU0UNCiAgICBudW1iZXJfc2VjdGlvbnM6IEZBTFNFDQotLS0NCg0KIyBBaW1zDQoNCiogVmlzdWFsaXphdGlvbiBvZiBhbnRpYm9keSBiaW5kaW5nIGZyb20gc2VydW0gDQoNCiMgQW5hbHlpcw0KDQpGdW5jdGlvbiBmb3IgdmlzdWFsaXphdGlvbjoNCg0KYGBge3J9DQpkcmF3X3BlcnNwXzNkIDwtIGZ1bmN0aW9uIChsb2dDLCBudSwgbG9neGksIGxvd2VyX3osIHVwcGVyX3opew0KICAgICANCiAjIyBEcmF3IHN1cmZhY2UgcGxvdA0KICAgICAjIHgtYXhpczogYW50aWJvZHkgY29uY2VudHJhdGlvbnMgKGxvZ1tBYl0pDQogICAgICMgeS1heGlzOiB0aXRyYXRpbmcgYmluZGluZyBzaXRlcyBpbW1vYmlsaXplZCBvbiB0aGUgc3VyZmFjZSAobG9nW0FnXSkNCiAgICAgIyB6LWF4aXM6IGNvbmNlbnRyYXRpb25zIG9mIGJvdW5kIGFudGlib2RpZXMgKGxvZ1tBYl1iKQ0KICAgICAjcGFyKGNleCA9IDAuOCkNCiAgICAgcGFyKGNleC5heGlzID0gMC43LCAgIyBzbWFsbGVyIHRpY2sgbGFiZWxzDQogICAgICAgICAgICAgICBjZXgubGFiICA9IDAuOSkgDQogICAgIHggPC0gc2VxKC0xLCAwLCBsZW5ndGgub3V0ID0gMjApDQogICAgIHkgPC0gc2VxKC02LCAtNCwgbGVuZ3RoLm91dCA9IDIwKQ0KICAgICB6X3N1cmZhY2UgPC0gb3V0ZXIoeCwgeSwgDQogICAgICAgICAgICAgICAgICAgICAgICBmdW5jdGlvbihhLCBiKSBsb2dDICsgbnUgKiBsb2coKDEgLyBudSkgLyAoMSArIGV4cCgtKGEpKSkpIC8gKDEgLSBudSkgKyBsb2coKDEgKyBudSkgLyAoMSArIG51ICogZXhwKC0oYiAtIGxvZ3hpKSkpKSAvIG51KQ0KICAgICB6X3N1cmZhY2Vbel9zdXJmYWNlIDwgbG93ZXJfeiB8IHpfc3VyZmFjZSA+IHVwcGVyX3pdIDwtIE5BIA0KICAgICBwbWF0IDwtIHBlcnNwKHgsIHksIHpfc3VyZmFjZSwNCiAgICAgICAgICAgICAgICAgICBjb2wgPSAiZ3JleTk5IiwgdGhldGEgPSA0NSwgcGhpID0gNDAsIHRpY2t0eXBlID0gImRldGFpbGVkIiwNCiAgICAgICAgICAgICAgICAgICB6bGltID0gYyhsb3dlcl96LCB1cHBlcl96KSwNCiAgICAgICAgICAgICAgICAgICB4bGFiID0gImxvZ1tBYl0iLCB5bGFiID0gImxvZ1tBZ10iLCB6bGFiID0gImxvZ1tBYl1iIiwNCiAgICAgICAgICAgICAgICAgICBib3JkZXIgPSAiZ3JleTcwIikNCiAgICAgDQogIyMgUmVkIGxpbmUgaW5kaWNhdGVzIHRoZSByZWxhdGl2ZSBjb25jZW50cmF0aW9uIChsb2coW0FiXWYvS0QpKQ0KICAgICB4X2hpZ2hsaWdodCA8LSAtbG9nKDEgLyBudSAtIDEsIGJhc2UgPSAxMCkNCiAgICAgel9oaWdobGlnaHQgPC0gb3V0ZXIocmVwKHhfaGlnaGxpZ2h0LCBsZW5ndGgoeSkpLCB5LA0KICAgICAgICAgICAgICAgICAgICAgICAgICBmdW5jdGlvbihhLCBiKSBsb2dDICsgbnUgKiBsb2coKDEgLyBudSkgLyAoMSArIGV4cCgtKGEpKSkpIC8gKDEgLSBudSkgKyBsb2coKDEgKyBudSkgLyAoMSArIG51ICogZXhwKC0oYiAtIGxvZ3hpKSkpKSAvIG51KVsxLCBdDQogICAgIHpfaGlnaGxpZ2h0W3pfaGlnaGxpZ2h0IDwgbG93ZXJfeiB8IHpfc3VyZmFjZSA+IHVwcGVyX3pdIDwtIE5BDQogICAgIHByb2plY3RlZF9saW5lIDwtIHRyYW5zM2QoeF9oaWdobGlnaHQsIHksIHpfaGlnaGxpZ2h0LCBwbWF0ID0gcG1hdCkgDQogICAgIGxpbmVzKHByb2plY3RlZF9saW5lJHgsIHByb2plY3RlZF9saW5lJHksIGNvbCA9ICJyZWQiLCBsd2QgPSAzKQ0KICAgICANCiAjIyBQdXJwbGUgbGluZSBpbmRpY2F0ZXMgZXBpdG9wZSBkZW5zaXR5IChsb2dbQWJdYikNCiAgICAgeF9saW5lIDwtIHJlcChtaW4oeCksIGxlbmd0aCh5KSkNCiAgICAgel9saW5lIDwtIHJlcChsb2dDLCBsZW5ndGgoeSkpDQogICAgIHByb2plY3RlZF9saW5lX2VkZ2UgPC0gdHJhbnMzZCh4X2xpbmUsIHksIHpfbGluZSwgcG1hdCA9IHBtYXQpDQogICAgIGxpbmVzKHByb2plY3RlZF9saW5lX2VkZ2UkeCwgcHJvamVjdGVkX2xpbmVfZWRnZSR5LCBjb2wgPSAicHVycGxlIiwgbHdkID0gMykNCiAgICAgDQogIyMgQmx1ZSBsaW5lIGluZGljYXRlcyB0aGUgYWZmaW5pdHkgKGxvZyhLRCkpDQogICAgIHpfaGlnaGxpZ2h0X3kgPC0gb3V0ZXIoeCwgcmVwKGxvZ3hpLCBsZW5ndGgoeCkpLA0KICAgICAgICAgICAgICAgICAgICAgICAgICAgIGZ1bmN0aW9uKGEsIGIpIGxvZ0MgKyBudSAqIGxvZygoMSAvIG51KSAvICgxICsgZXhwKC0oYSkpKSkgLyAoMSAtIG51KSArIGxvZygoMSArIG51KSAvICgxICsgbnUgKiBleHAoLShiIC0gbG9neGkpKSkpIC8gbnUpWywgXQ0KICAgICBwcm9qZWN0ZWRfbGluZV95IDwtIHRyYW5zM2QoeCwgcmVwKGxvZ3hpLCBsZW5ndGgoeCkpLCB6X2hpZ2hsaWdodF95WywgMV0sIHBtYXQgPSBwbWF0KQ0KICAgICBsaW5lcyhwcm9qZWN0ZWRfbGluZV95JHgsIHByb2plY3RlZF9saW5lX3kkeSwgY29sID0gImJsdWUiLCBsd2QgPSAzKQ0KfQ0KYGBgDQoNClN1cmZhY2UgZGlzcGxheSBmb3IgdGhlIGNoYXJhY3Rlcml6YXRpb24gb2YgYW50aWJvZHkgYmluZGluZyB0byBTQVJTLUNvVi0yIHNwaWtlIHByb3RlaW4gaW4gc2VydW0NCg0KYGBge3IsIGZpZy5oZWlnaHQ9NH0NCmRyYXdfcGVyc3BfM2QobG9nQyA9IC04LA0KICAgICAgICAgICAgICBudSA9IDAuMywNCiAgICAgICAgICAgICAgbG9neGkgPSAtNSwNCiAgICAgICAgICAgICAgbG93ZXJfeiA9IC0xMSwNCiAgICAgICAgICAgICAgdXBwZXJfeiA9IC01KQ0KYGBgDQoNCg0K
